# Distinct Differences in the Expansion and Phenotype of TB10.4 Specific CD8 and CD4 T Cells after Infection with *Mycobacterium tuberculosis*

**DOI:** 10.1371/journal.pone.0005928

**Published:** 2009-06-16

**Authors:** Truc Thi Kim Thanh Hoang, Anneline Nansen, Sugata Roy, Rolf Billeskov, Claus Aagaard, Tara Elvang, Jes Dietrich, Peter Andersen

**Affiliations:** 1 Department of Infectious Disease Immunology, Statens Serum Institut, Copenhagen, Denmark; 2 Department of Immunopharmacology, Novo Nordisk, Måløv, Denmark; Tulane National Primate Research Center, United States of America

## Abstract

**Background:**

Recently we and others have identified CD8 and CD4 T cell epitopes within the highly expressed *M. tuberculosis* protein TB10.4. This has enabled, for the first time, a comparative study of the dynamics and function of CD4 and CD8 T cells specific for epitopes within the same protein in various stages of TB infection.

**Methods and Findings:**

We focused on T cells directed to two epitopes in TB10.4; the MHC class I restricted epitope TB10.4 _3–11_ (CD8/10.4 T cells) and the MHC class II restricted epitope TB10.4 _74–88_ (CD4/10.4 T cells). CD4/10.4 and CD8/10.4 T cells displayed marked differences in terms of expansion and contraction in a mouse TB model. CD4/10.4 T cells dominated in the early phase of infection whereas CD8/10.4 T cells were expanded after week 16 and reached 5–8 fold higher numbers in the late phase of infection. In the early phase of infection both CD4/10.4 and CD8/10.4 T cells were characterized by 20–25% polyfunctional cells (IL-2^+^, IFN-γ^+^, TNF-α^+^), but whereas the majority of CD4/10.4 T cells were maintained as polyfunctional T cells throughout infection, CD8/10.4 T cells differentiated almost exclusively into effector cells (IFN-γ^+^, TNF-α^+^). Both CD4/10.4 and CD8/10.4 T cells exhibited cytotoxicity in vivo in the early phase of infection, but whereas CD4/10.4 cell mediated cytotoxicity waned during the infection, CD8/10.4 T cells exhibited increasing cytotoxic potential throughout the infection.

**Conclusions/Significance:**

Our results show that CD4 and CD8 T cells directed to epitopes in the same antigen differ both in their kinetics and functional characteristics throughout an infection with *M. tuberculosis*. In addition, the observed strong expansion of CD8 T cells in the late stages of infection could have implications for the development of post exposure vaccines against latent TB.

## Introduction

*Mycobacterium tuberculosis* (*M.tb*) continues to be a major threat to global health and immense efforts are currently invested in an attempt to understand the immune mechanisms involved in controlling this chronic disease. CD4 T cells are induced early during the acute phase whereas CD8 T cells have been reported to expand in the later stages of the infection [1], [2]. However, the study of the dynamic development of antigen specific T cells at the clonal level has been complicated by the fact that CD8 T cell epitopes have only recently been identified within *M.tb* antigens. Therefore, examining the involvement of CD8 T cells in the defense against an infection with *M.tb* has in large part been based on the study of bulk CD8 T cells as in the study performed by Lazarevic et al. [1]. Recent studies have identified new CD8 T cell epitopes within *M.tb* derived proteins, such as CFP-10 and TB10.4 [3]–[8]. TB10.4 is a low molecular mass protein that belongs to the ESAT-6 family and was found to be highly immunogenic and to confer protection against an aerosol challenge with *M.tb* when administered to mice in a subunit vaccine composed of either TB10.4 alone or TB10.4 fused to another immune dominant *M.tb* protein, Ag85B [9], [10]. Several T cell epitopes have been identified within TB10.4, namely the MHC class I restricted epitopes; the H2-*K^b^* TB10.4 _3–11_, H2-*K^d^* TB10.4 _20–28_ and the MHC class II restricted epitope H2-*K^d^* TB10.4 _74–88_ [3], [4], [11], [12].

With the recently emerging novel information on CD4 and CD8 T cells within the TB10.4 antigen it has now become possible to study the dynamics of the emergence, expansion and contraction of CD4 and CD8 T cell populations directed to epitopes derived from the same *M.tb* protein. This represents a unique opportunity to study the dynamic development of these subsets without the interference imposed by a temporal shift in the expression of different *M.tb* proteins during the course of a TB infection. In the present study we therefore focused on T cells directed against the two identified epitopes in TB10.4; the MHC-class I restricted H2-*K^b^* TB10.4 _3–11_ CD8 epitope [3] and the MHC class II restricted H2-*K^d^* TB10.4 _74–88_ CD4 epitope [11]. As these epitopes are restricted to different haplotypes we used the CB6F1 hybrid (BALB/c×C57BL/6) which enabled us to study both T cell populations in one mouse strain in terms of dynamics as well as functional and phenotypic changes during a persistent *M.tb* infection. We found that the dynamics of expansion, contraction and functional characteristics differed markedly for CD8/10.4 and CD4/10.4 T cells throughout a TB aerosol infection.

## Results

### The dynamic development of CD4 and CD8 responses to TB10.4 during TB infection

**Figure 1:**
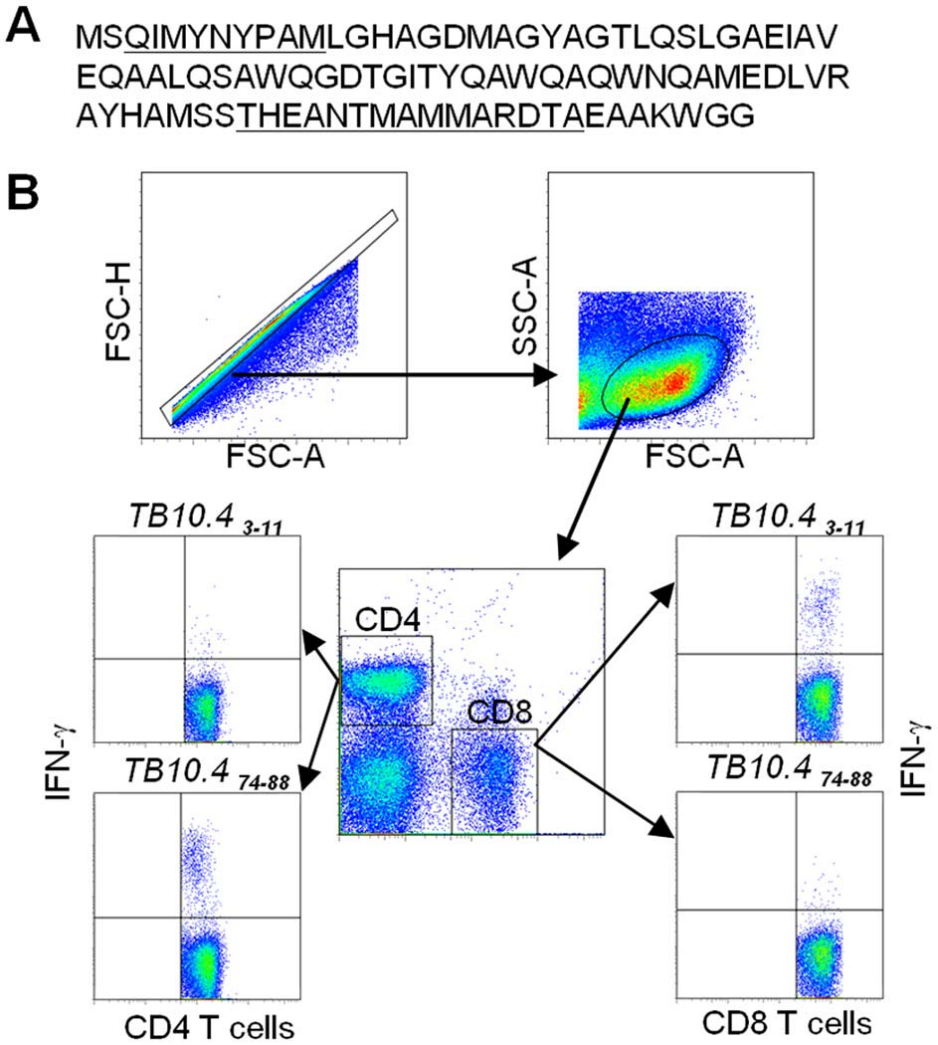
The TB10.4 CD4 and CD8 T cells epitopes that are recognized by CB6F1 mice (BALB/c×C57BL/6). (A) the amino acid sequence of the TB10.4 protein with the CD4 and CD8 T cell epitopes underlined. (B) Lung lymphocytes from mice infected at week six after aerosol infection were stimulated in vitro with either the TB10.4 _3–11_ (QIMYNYPAM) or the TB10.4 _74–88_ (THEANTMAMMARDT) for 6 hours before being assessed for IFN-γ production by flow cytometry. A gating sequence was applied to the data to determine the frequencies of IFN-γ positive CD4 and CD8 T cells. The rationale for the FSC-H vs. FSC-A gating is to capture only singlet particles and eliminate doublets that may occur as a result of e.g. cells sticking together.

**Figure 2:**
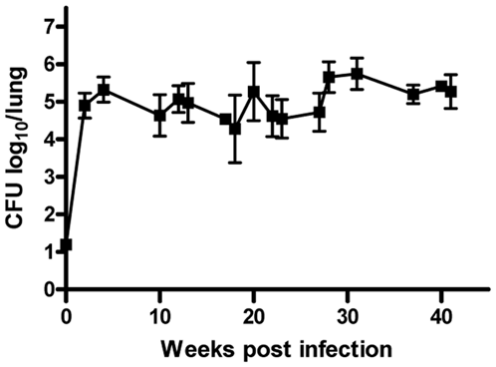
The bacterial load in the lungs throughout infection shown as Log10 CFU. Mice were challenged by the aerosol route with virulent *M. tuberculosis*. At the indicated timepoints 3–6 mice were killed and the bacterial burden (CFU) was measured in the lung.

**Figure 3:**
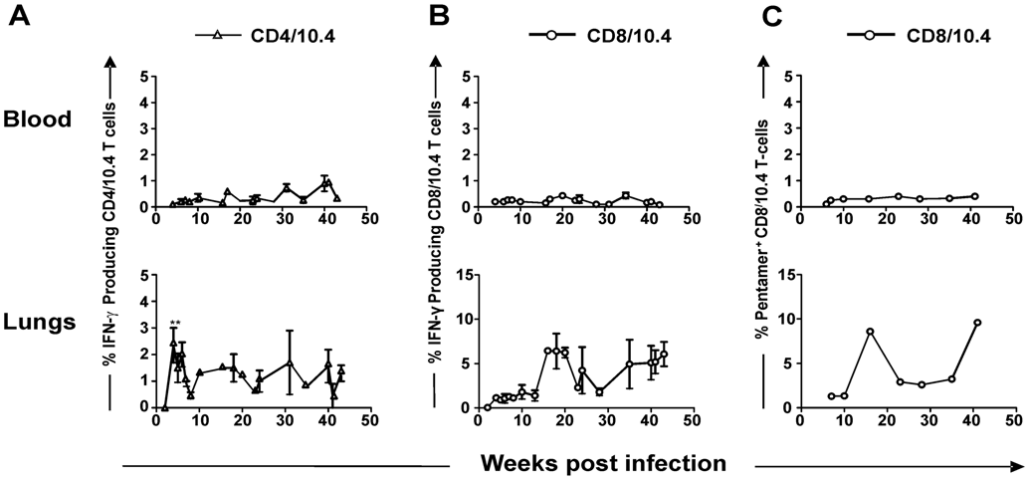
The kinetics of CD4/10.4 and CD8/10.4 following aerosol infection with *M.tb*. (A and B) CB6F1 mice were infected by the aerosol route with virulent *M.tb Erdman* and lymphocytes were obtained from lungs or blood and then stimulated with TB10.4 _3–11_ or TB10.4 _74–88_ for assessment of IFN-γ production by FACS analysis. Frequencies represent IFN-γ production out of total T cells. Background staining from cells stimulated with medium alone has been deducted (Background <0.5%). Each time point represents the mean from at least three individual mice±standard error of the mean (SEM). (C) Lung or blood cells were stained directly *ex vivo* with H2-*K^b^* pentamer loaded with IMYNYPAM. Background staining from naïve mice has been deducted. Each time point consists of data from a pool of 3–6 mice. (**p<0.01, Student's t-test).

Anti-TB10.4 _3–11_ CD8 T cells and anti-TB10.4 _74–88_ CD4 T cells (hereafter called CD8/10.4 and CD4/10.4 cells) represent a significant proportion of the T cells induced by infection with *M.tb* [3]. To study and compare the dynamic development of these T cells, CB6F1 (BALB/c×C57BL/6) mice were infected by the aerosol route with *M.tb Erdman* and the T cell immune response against TB10.4 _74–88_ and TB10.4 _3–11_ was analyzed at different time points following infection. Epitope recognition was assessed using TB10.4 _74–88_ and TB10.4 _3–11_ peptides for in vitro stimulation of lymphocytes from infected mice and the frequency of CD4/10.4 or CD8/10.4 T cells out of all T cells following 6 hour stimulation with antigen was analyzed by flow cytometry (calculation shown in materials and methods) (Fig. 1). The CFU levels in the lung showed an increase up to week 4–5 post infection where after the CFU levels did not change significantly throughout the course of infection (Fig. 2). In terms of the CD4/10.4 and CD8/10.4 cells the dynamic development of priming, expansion and contraction of these cells followed different patterns (Fig. 3). In the spleen and blood, both CD4/10.4 and CD8/10.4 cells could be detected, but the frequencies out of all lymphocytes, were around 1%, and not as high as seen in the lung. In the lungs, CD4/10.4 cells expanded rapidly and reached around 3% of all T cells (5.5% of all CD4 T cells, data not shown) 4–6 weeks after infection, which represented a significant increase in CD4/10.4 cell numbers compared to week 0 post infection (student's T test, p<0.05). The percentage then declined to approximately 1.5% of all T cells and it stayed at that level for the rest of the experiment (until week 43) (Fig. 3A). In contrast, CD8/10.4 displayed a delayed kinetic and this T cell population reached its initial peak as late as week 15 post-infection where 6.5% of all T cells (and 18% of all CD8 T cells, data not shown) recognized this epitope. This was followed by a contraction from week 22–27 (to around 2%) after which CD8/10.4 cells again stabilized at a level between 5–6% of all T cells (and 18% of all CD8 T cells, data not shown) (Fig. 3B). The accelerated expansion of CD4/10.4 was confirmed by ELISPOT at week 6 where TB10.4 _74–88_ stimulation induced up to 7 fold more spot forming (CD4/10.4) cells than stimulation with the CD8 epitope TB10.4 _3–11_ (data not shown). Staining the cells with the *H-2K^b^*/TB10.4 pentamer that specifically identified CD8/10.4 cells, confirmed the kinetic pattern observed after peptide stimulation (Fig. 3C) and the similar percentages obtained by pentamer and IFN-γ staining indicated that this CD8 T cell population expressed IFN-γ throughout the observation period. Thus, following infection the frequency of CD4 and CD8 T cells specific for epitopes both encoded within the TB10.4 molecule, followed distinct patterns.

### Activation state of CD8/10.4 and CD4/10.4 T cells throughout infection

**Figure 4:**
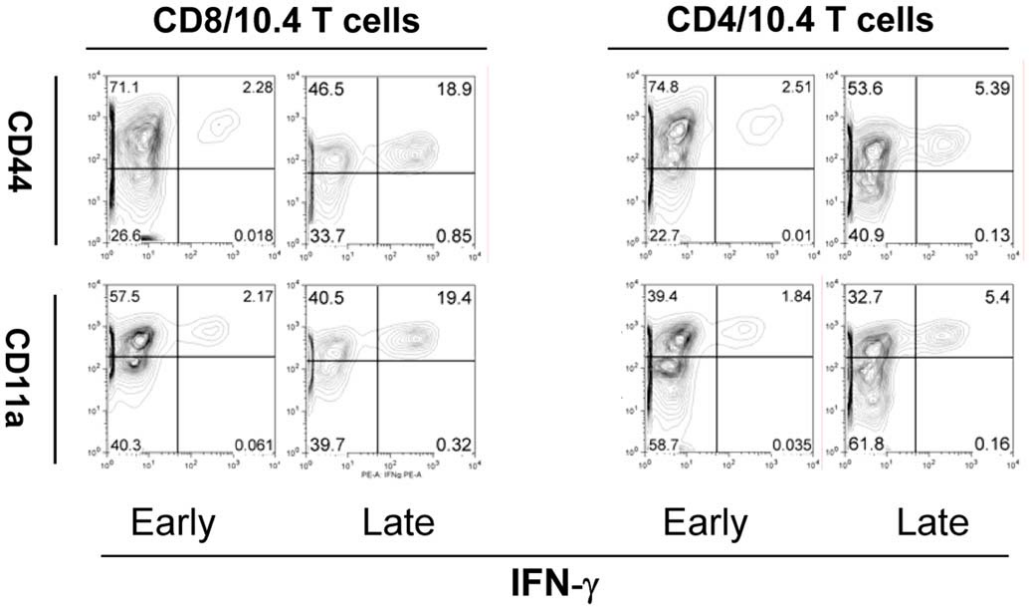
Phenotypic analysis of CD4/10.4 and CD8/10.4 during a persistent infection. Lung lymphocytes from mice sacrificed at different stages of infection (Early: week 6; late: week 40) were stimulated with (A) TB10.4 _3–11_ or (B) TB10.4 _74–88_ prior to staining for the surface markers CD44, CD11a and intracellularly for IFN-γ. Samples were then examined by flow cytometry. FACS plots are shown for one mouse, representative of 3–4 mice.

To characterize the phenotype of CD8/10.4 and CD4/10.4 T cells, cells isolated from the lungs during infection were stimulated in vitro with TB10.4 _74–88_ and TB10.4 _3–11_ and analyzed by flow cytometry for expression of CD4, CD8, CD44, CD11a and IFN-γ. Based on the dynamic changes in the size of the CD8/10.4 and CD4/10.4 T cells (Fig. 3), we compared the phenotype of the epitope specific cells during the different stages of the infection i.e. the early stage (week 6) and the late stage (week 40). At both time points the majority of the IFN-γ producing CD4 and CD8 T cells were CD44^high^ and thus resembled a typical effector phenotype. The same cells also expressed low levels of CD45RB and CD62L confirming their effector phenotype, and at other time points throughout the infection this staining profile did not change (data not shown). The expression of these markers was not significantly changed during the in vitro stimulation itself (data not shown). As the percentages indicated in the figure are out of the CD8 or CD4 T cell population, they are higher compared to the percentages in figure 3 which are out of the total number of T cells. As expected, both the CD8/10.4 and CD4/10.4 T cells expressed CD11a which has been shown to be an important homing marker to the lungs for T cells during an infection with *M.tb* [13]. Thus, both CD4/10.4 and CD8/10.4 T cells displayed an effector phenotype and no significant phenotypic changes, based on these surface markers, were seen for the two T cell populations over the course of the infection (Fig. 4).

### Cytokine expression by CD8/10.4 and CD4/10.4 T cells throughout infection

**Figure 5:**
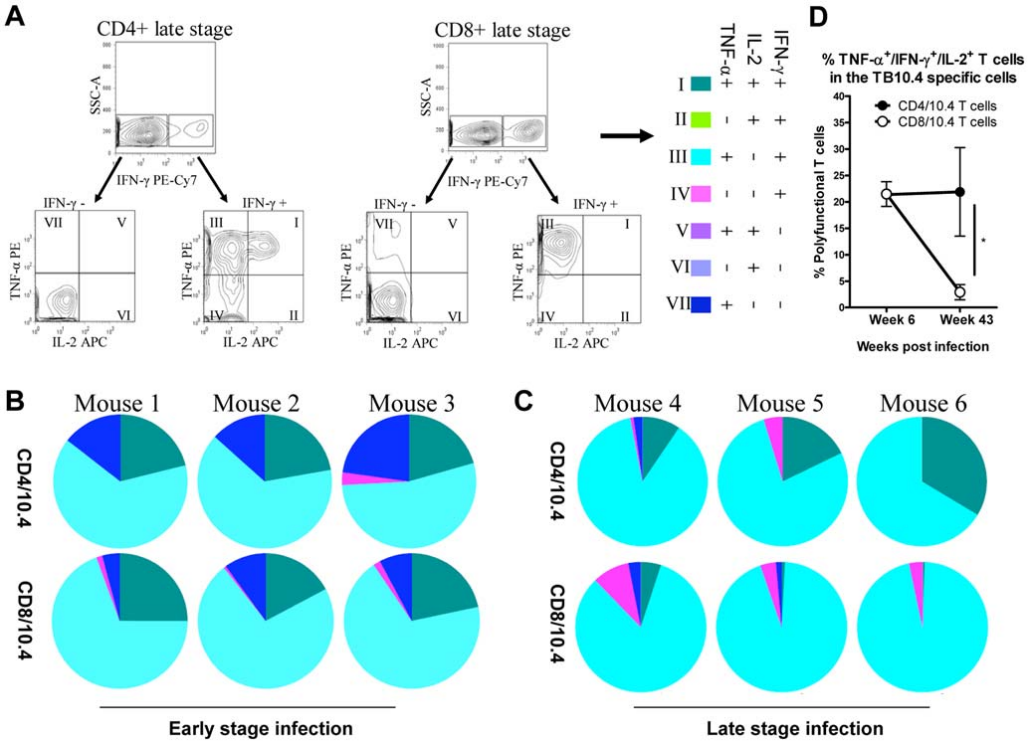
Changes in cytokine profiles of CD4/10.4 and CD8/10.4 T cells during a chronic infection. Cells from infected lungs were stimulated with TB10.4 _3–11_ or TB10.4 _74–88_ prior to staining with anti-CD4, -CD8, -IFN-γ, -TNF-α and –IL-2. (A) Cytokine profiles of CD4/10.4 was determined by first dividing the CD4 T cells into IFN-γ positive (+) or IFN-γ negative (−) cells. Both the IFN-γ+ and IFN-γ- cells were analyzed with respect to the production of TNF-α and IL-2. The numbers in the quadrant gates of the plots denominates each distinct population based on their cytokine production and is color coded as shown. To illustrate the gating sequence two examples from a late infection stage are shown. (B and C). The pie-charts illustrate the relative contribution of the different cytokine T cell populations to the total CD4/10.4 population and data is depicted for both an early and a late time point, for 3 mice at each time point. Background (>0.5%) has been deducted. (D) The proportion of IFN-γ^+^ TNF-α^+^ IL-2^+^ within the CD4/10.4 or CD8/10.4 T cells populations in the early stage compared to the late stage of infection.

We next looked at the co-expression of multiple cytokines in CD8/10.4 and CD4/10.4 T cells. Cells from infected lungs, taken at week 6 or week 43 post infection, were stimulated with TB10.4 _3–11_ or TB10.4 _74–88_ prior to staining with anti-CD4, -CD8, -IFN-γ, -TNF-α and –IL-2. The results showed that TNF-α/IFN-γ effector cells constituted the major population observed among both CD4 and CD8/10.4 T cells (Fig. 5A–C), reflecting an ongoing stimulation in the *M.tb* infected animals, and in agreement with the results shown in figure 4. However, in terms of polyfunctionality, i.e. the co-expression of multiple cytokines such as TNF-α, IFN-γ and IL-2, we observed a difference between the two T cell populations as the infection progressed. Thus, at the late time point there was still a significant proportion of the CD4/10.4 cells that were polyfunctional in contrast to the CD8/10.4 cells (Fig. 5B and C). For the CD8/10.4 T cell population the proportion of TNF-α, IFN-γ and IL-2 co-expressing cells decreased from around 25% during the early timepoint to 0.5% during the late timepoint. However, the proportion of polyfunctional CD4/10.4 T cells did not change significantly despite the ongoing infection (Fig. 5D). Taken together, these results showed that in contrast to the CD8/10.4 T cells, a considerable proportion of the CD4/10.4 T cells were maintained as polyfunctional T cells.

### Cytotoxic potential of CD8/10.4 and CD4/10.4 T cells throughout infection

**Figure 6:**
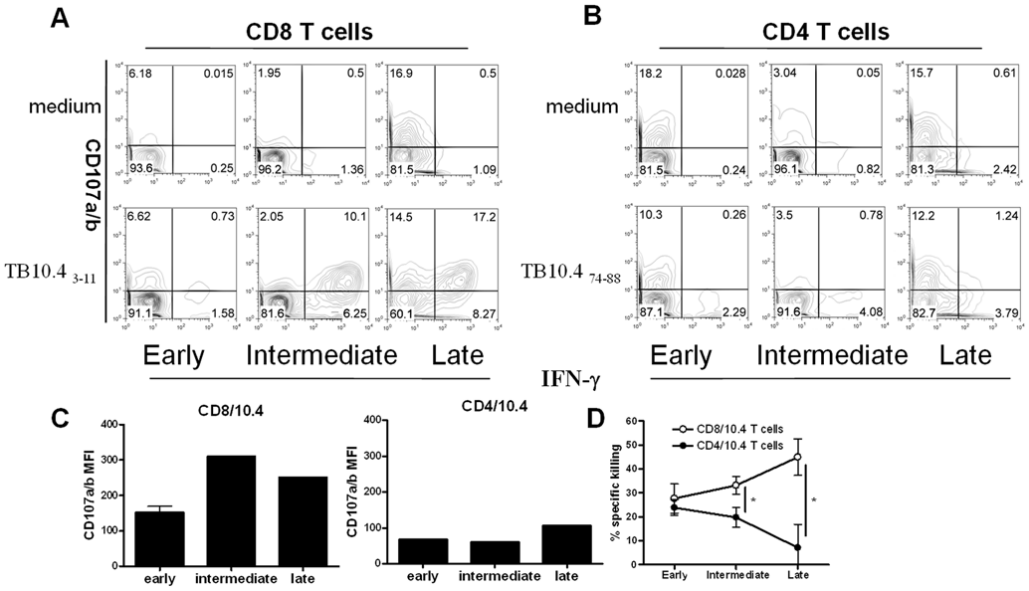
Functional characterization of the CD4/10.4 and CD8/10.4 T cells at different stages of infection. (Early: week 6; Intermediate: week 16, late: week 40) (A and B) Lung cells from infected mice were stimulated in vitro with TB10.4 _3–11_ or TB10.4 _74–88_ (lower panel) or left non stimulated (“Media”, upper panel) in the presence of αCD107a/b and stained with αIFN-γ and αCD8 (A), or αCD4 (B), antibodies (C) CD107a/b MFI of the IFN-γ positive cells following in vitro stimulation with TB10.4 _3–11_ or TB10.4 _74–88_. (D) The specific lysis of TB10.4 _3–11_ or TB10.4 _74–88_ loaded cells was determined in an in vivo cytotoxicity assay. Unloaded splenocytes (CFSE^low^) and TB10.4 _3–11_ or TB10.4 _74–88_ loaded splenocytes (CFSE^high^) from naïve mice were transferred into infected mice. The amount of splenocytes killed in vivo by cytotoxic T cells specific for either TB10.4 _3–11_ or TB10.4 _74–88_ was observed as a reduction in the CFSE^high^ population and a percent specific lysis was calculated. A value of *P*<0.05 (students paired t-test) was considered significant and is shown by *.

We also compared the functional capabilities of the CD4/10.4 and CD8/10.4 cells in terms of cytotoxicity. We first investigated CD107a/b, which is a known marker for degranulation. A clear change over time in the expression of CD107a/b was observed. On CD8/10.4 T cells expression of CD107a/b increased up to ∼70% during the intermediate and late chronic phase (Fig. 6A). In contrast, we found only a minor increase in the percentage of CD4/10.4 IFN-γ^+^ CD107a/b^+^ cells during the infection (Fig. 6B). This difference in CD107a/b expression was also reflected in the CD107a/b MFI which showed a higher increase on CD8/10.4 IFN-γ^+^ T cells than on CD4/10.4 IFN-γ^+^ T cells as the infection progressed (Fig. 6C). As these results indicated an increased cytotoxic potential of CD8/10.4 T cells in the late stages of infection, we also compared the in vivo capability of CD8/10.4 and CD4/10.4 T cells to eliminate target cells presenting the specific epitope in infected mice throughout infection. We used the in vivo cytotoxicity assay where CFSE labeled splenocytes from naïve mice, unpulsed or pulsed with either TB10.4 _3–11_ or TB10.4 _74–88_ were adoptively transferred into infected mice. Thereafter, peptide specific lysis of the transferred cells was investigated by flow cytometric analysis of infected recipient lung cells. In the early phase of the infection we observed approximately 30% specific killing of both TB10.4 _3–11_ or TB10.4 _74–88_ loaded target cells, demonstrating that both CD8/10.4 and CD4/10.4 T cells possessed cytotoxic potential (Fig. 6D). However, in the later phase of the infection, and in particular during the late chronic phase, a significant increase in the clearance of TB10.4 _3–11_ pulsed target cells was observed indicating a qualitative change of the CD8/10.4 T cells over time (Fig. 6D). In contrast to the increased cytotoxic potential of CD8/10.4 T cells over time, CD4/10.4 T cells initially exhibited cytolytic activity (24% specific killing) that however decreased to 7% at the late chronic stage (Fig. 6D). Thus, both in terms of both polyfunctionality and cytoxicity CD4/10.4 and CD8/10.4 T cells showed marked differences.

## Discussion

Numerous reports have focused on the characterization of T cells primed during infection with *M.tb*. The majority of these studies have been based on the description of bulk T cells and has not addressed the characteristics of single epitope specific T cells [1], [14], [15]. However recently, due to the discovery of new *M.tb* T cell epitopes, a number of laboratories have focused on the characterization of single T cell clones elicited following an infection. Thus, a report on tracking M.tb72F epitope specific CD8 T cells induced after infection showed that the CD8 T cells were present in significant numbers over the course the infection and that the CD8 T cells appeared to change from an effector phenotype to a more T cell memory-like phenotype [6]. Other laboratories have identified *M.tb* induced CD8 T cells specific for either TB10.4 or CFP10 in the C57BL/6 and BALB/c mouse model and demonstrated that they exhibit cytolytic activity [3], [5]. However, only recently have both class I and II restricted epitopes within the same antigen been identified thereby enabling a study of the emergence, expansion and contraction of specific CD4 and CD8 T cells during the course of a TB infection without the interference imposed by a potential temporal shift in the expression of different *M.tb* antigens during the course of a TB infection.

In the present study we provided a functional and phenotypical characterization of TB10.4 epitope specific CD4 and CD8 T cells during a long term chronic infection. TB10.4 is part of the ESAT-6 family of proteins and is highly expressed throughout an infection with *M.tb* [10], [16]. We focused on the TB10.4 CD4 epitope THEANTMAMMARDT (TB10.4 _74–88_) and the CD8 epitope QIMYNYPAM (TB10.4 _3–11_). These epitopes have been shown to elicit strong responses leading to high numbers of TB10.4 _3–11_ specific CD8 T cells in C57BL/6 or TB10.4 _74–88_ specific CD4 T cells in BALB/c mice [3], [11], [17].

In agreement with previous studies, aerosol infection with *M.tb* induced substantial amounts of TB10.4 specific CD8 and CD4 T cells in the lungs. This was observed after measuring IFN-γ positive T cells following in vitro stimulation with the epitopes or after staining the CD8 T cells ex vivo with the H2-*K^b^* pentamer (Fig. 1 and 3). In the acute phase of infection (<6 weeks) the CD4/10.4 T cells were recruited and expanded at the site of infection in an accelerated fashion (Fig. 3) that resulted in 2–9 times more CD4/10.4 T cells at week 6 of infection (measured by FACS or ELISPOT, (fig. 3, and data not shown). In the later stages of infection CD4/10.4 T cells were maintained at constant levels and accounted for 1–3% of the total T cell population throughout infection (up till 43 weeks after infection, Fig. 3). CD8/10.4 cells for comparison were greatly expanded in the later stages of infection (week 16 and 35), in agreement with a previous study conducted on bulk CD8 T cells [1]. Thus, following the initial acute phase we observed an increase in the frequency of CD8/10.4 T cells from week 16 to week 20 where up to 6.5–9% of all T cells were specific for TB10.4 _3–11_ (Fig. 3B and C). Thereafter, we observed a decline in the number of CD8/10.4 cells to between 2 and 4% between week 22–27 post infection. This was followed by an increase in numbers with levels above 5% post week 35 of infection (Fig. 3B and C). It is interesting that the major expansion of CD8 T cells occurred in the later stages of infection. This implies that these cells may serve an important role at this stage of infection, which could have implications for the development of post exposure vaccines against latent TB. Indeed, previous experiment in a mouse model for latent TB showed that depleting of CD8 T cells led to reactivation of latent TB [18].

It has been suggested that such a dynamic behavior could reflect the fluctuating responsiveness of the immune system to the periodic and transient bursts of mycobacterial replication inside infected lungs and a fine-tuning of the response to control the infection without inducing substantial pathology [1]. A recent study suggested that this dynamic fluctuation of T cell numbers occurred simultaneously for both subsets, but studied the dynamics of the overall T cell subsets and not T cells at an antigen specific level [1]. In contrast, our study was based on the tracking of epitope specific CD4 and CD8 T cells, and we observed a clear difference in the frequencies of the TB10.4 specific CD8 and CD4 T cells (Fig. 3), in that CD4/10.4 dominated the early stages and CD8/10.4 cells the intermediate and late stages. It could be speculated that in the early stages the bacteria are primarily taken up by professional APC's and via the phagosomes directed to the MHC-II processing pathway leading to a preferential priming of CD4 T cells. It is however important in this context to emphasize that the difference we observe is a difference in quantity and kinetics and does not reflect a complete lack of MHC-I presentation as we also find detectable levels of CD8/10.4 T cells in the early phase of infection although at a lower level than CD4/10.4 T cells. However, in the later stages of infection (>15 weeks), a change in the processing of antigen occurs that favor presentation on MHC-I and expansion of CD8/10.4. This change could involve increased transfer of antigen from phagosomes into the MHC class I pathway in heavily infected DCs [19], [20] or increased apoptosis of antigen loaded macrophages and release of antigen material or mycobacteria for subsequent uptake and cross-priming in either CD8^+^ DC's [21] or neutrophils [19], [20], [22]. Neutrophils in particular are an interesting possibility as they have been demonstrated to be a very efficient source of cross-priming in vivo and are abundant in TB granulomas in the late or chronic phase of infection [22], [23]. Finally, as the bacterial numbers increase, infection of non-APC's, that primarily present antigens on MHC-I, may also increase and thus favor expansion of CD8 T cell numbers. Such cells could be the epithelial cells, which have indeed been shown to be infected by *M.tb* during a chronic infection [24].

A few other recent studies have reported tracking of specific CD8 T cells. Thus, as mentioned above, in a recent study using a class I tetramer reagent to track M.tb72F antigen-specific CD8 T cells (GAPINSATAM) in the lungs of infected mice up to day 100 post infection, a different and less dynamic kinetic pattern was observed. The frequency of GAPINSATAM specific CD8 T cells gradually increased up till day 30 post infection (∼week 4) and then declined until day 100 (∼week 14) [6]. It may be that the different findings from this study, compared to our results, relate to a differential antigenic expression of the mycobacterial proteins TB10.4 and M.tb72F or the processing of the epitopes QIMYNYPAM and GAPINSATAM. Other long term studies of T cell populations have been performed recently in our and other laboratories. Thus, Billeskov et al. demonstrated that TB10.4 _3–11_ specific CD8 T cells comprised a large part of the CD8 T cell population in the lungs of infected C57BL/6 mice at 50 weeks post infection [3] and Kamath et al. showed that at 32 weeks post infection there was approximately 16% TB10.4 _20–28_ specific CD8 T cells in the lungs of infected BALB/c mice [5]. However, as these studies did not, to the same degree as the present study, include a kinetic analysis of both CD4 and CD8 T cells specific for the same protein, the main conclusions were that TB10.4 or CFP10 CD8 T cells dominate in the late phases of infection, and a distinct kinetic pattern that differed from that of the CD4 T cells against the same protein, was not described.

Throughout the infection all TB10.4 _3–11_ and TB10.4 _74.88_ epitope specific CD8 and CD4 T cells displayed an effector phenotype in agreement with a previous study which also showed that both CD4 and CD8 T cell bulk populations in the lungs of chronically infected mice expressed a cell surface phenotype consistent with that of effector T cells (Fig. 4 and [25]). All IFN-γ^+^ cells expressed CD11a implicating the significance of this particular integrin as confirmed by the increased susceptibility to aerosol *M.tb* infection in CD11a gene knockout mice [13]. Comparing the expression of TNF-α, IFN-γ and IL-2 among CD4/10.4 and CD8/10.4 T cells at an early and late time point showed that whereas both populations contained polyfunctional T cells at early stages of infection, only CD4/10.4 maintained a substantial part of the cell population as polyfunctional T cells (Fig. 5A–C). In contrast, CD8/10.4 T cells all developed into terminally differentiated effector cells which is in agreement with the observations from persistent viral infections where chronicity is associated with exhaustion, loss of both CD8 function and polyfunctionality [26], [27]. Interestingly, the presence of polyfunctional T cells have been shown to correlate with protective immunity against infections such as *Leishmania major*, and to form the basis for a long lived memory response [28], indicating that this subset of T cells may also be important for the protection against infection with *M.tb*, or reactivation of latent TB. CD4/10.4 and CD8/10.4 also differed in terms of cytotoxicity. We first looked at the degranulation marker CD107a/b which have been demonstrated to correlate with cytotoxicity [29], [30]. During the acute phase, 30% of the TB10.4 _3–11_ specific CD8 T cells expressed CD107a/b as opposed to the ∼10% expressed by the TB10.4 _74–88_ specific CD4 T cells. As the disease progressed no significant increase in CD107a/b expression was observed within the TB10.4 _74–88_ specific CD4 T cell population in contrast to the TB10.4 _3–11_ specific CD8 T cells where the numbers increased to approximately 70% of all IFN-γ producing TB10.4 _3–11_ cells (17.2%/(17.2%+8.27%), see also figure 6) indicating that these cells were potentially more cytotoxic (Fig. 6). The ability of the TB10.4 specific CD8 T cell to perform cytolysis of peptide pulsed target cells in vivo was indeed confirmed in the in vivo cytotoxicity assay where the killing of TB10.4 _3–11_ pulsed target cells increased with time in contrast to the killing of TB10.4 _74–88_ pulsed target cells which decreased with time (Fig. 6D). Interestingly, during the early and intermediate phase of infection CD4/10.4 displayed significant cytolytic activity, despite only a minor expression of CD107a/b. This indicated that CD4/10.4 T cells may exert their cytotoxic capabilities through other pathways besides degranulation, such as the Fas/FasL pathway [31]. However, at later stages of the infection CD4/10.4 cells gradually lost this cytotoxic function in contrast to the CD8/10.4 cells.

In conclusion, this study shows that CD4 and CD8 T cells specific for epitopes on the same *M.tb* antigen display markedly different dynamic patterns in terms of expansion, contraction and functional properties throughout an *M.tb* infection. Our results also indicate that whereas the function of CD4/10.4 and CD8/10.4 cells may be partially overlapping in the early stages of infection (judged by the small differences in polyfunctionality, CD107a/b expression, and in vivo cytoxicity), at later time points these cells showed significant differences in these markers suggesting more disparate effector functions of the subsets in the late stages of infection.

## Materials and Methods

### Animal handling

Studies were performed with 6–8-week-old female CB6F1 (BALB/c×C57BL/6) from Harland Netherlands. Non-infected mice were housed in cages in appropriate animal facilities at Statens Serum Institut. Infected animals were housed in cages contained within laminar flow safety enclosures (Scantainer from Scanbur, Denmark) in a separate biosafety level 3 facility. All mice were fed radiation sterilized 2016 Global Rodent Maintenance diet (Harlan, Scandinavia) and water *ad libitum*. All animals were allowed a 1-week rest period after delivery before the initiation of the experiments. The handling of mice were conducted in accordance with the regulations set forward by the Danish Ministry of Justice and animal protection committees by Danish Animal Experiments Inspectorate permit 2004-561-868 of 01-07-2004. This was in compliance with European Community Directive 86/609 and the U.S. Association for Laboratory Animal Care recommendations for the care and use of laboratory animals. All animal handling was done at Statens Serum Institut by authorized personnel.

### Bacteria

*M. tuberculosis Erdman* was grown at 37°C in suspension in Sauton medium (BD Pharmingen) enriched with 0.5% sodium pyruvate 0.5% glucose 0.2% Tween 80. All bacteria were stored at −80°C in growth medium at ∼5×10^8^ CFU/ml. Bacteria were thawed, sonicated, washed and diluted in phosphate-buffered saline (PBS). All bacterial work was performed at the Statens Serum Institut by authorized personnel.

### Antigens

Synthetic overlapping peptides (18-mers and 9-mers) covering the complete primary structure of TB10.4 along with the TB10.4 peptides TB10.4 _3–11_ QIMYNYPAM and TB10.4 _74–88_ THEANTMAMMARDT were synthesized by standard solid-phase methods on a SyRo peptide synthesizer (MultiSynTech, New England) at the JPT Peptide Technologies (Berlin, Germany), or at Schafer-N (Copenhagen, Denmark). Peptides were lyophilized and stored dry at −20°C until reconstitution in PBS.

### Experimental infections

Upon challenge by the aerosol route, the animals were infected with either a low dose ∼50 CFU or high dose ∼100–150 CFU of *M.tb Erdman*/mouse with an inhalation exposure system (Glas-Col, Indiana,USA). The numbers of bacteria in the spleen or lung were determined by serial 3-fold dilutions of individual whole-organ homogenates in duplicate on 7H11 medium supplemented with PANTA™ (Becton Dickinson, San Diego, USA). Colonies were counted after 2–3 wk of incubation at 37°C.

### Lymphocyte cultures

Peripheral blood mononuclear cells (PBMCs) were purified on a density gradient of mammal lympholyte® cell separation media (Cedarlane Laboratories Inc., Canada). Splenocyte cultures were obtained by passage of spleens through a metal mesh followed by two washing procedures using RPMI. Lung lymphocytes were obtained by passage of lungs through a 100 µm nylon cell strainer (BD Pharmingen, USA) followed by two washing procedures using RPMI. Cells in each experiment were cultured in sterile microtiter wells (96-well plates; Nunc, Denmark) containing 2−10×10^5^ cells in 200 µl of RPMI 1640 supplemented with 1% (v/v) premixed penicillin-streptomycin solution (Invitrogen Life Technologies), 1 mM glutamine, and 10% (v/v) fetal calve serum (FCS) at 37°C/5% CO_2_.

### Flow cytometric analysis

Intracellular cytokine staining procedure: Cells from blood, spleen or lungs of mice were stimulated for 1–2 h with 2 µg/ml Ag at 37°C and subsequently incubated for 5 h at 37°C with 10 µg/ml brefeldin A (Sigma-Aldrich, Denmark) at 37°C. Fc receptors were blocked with 0.5 µg/ml anti-CD16/CD32 mAb (BD Pharmingen, USA) for 10 minutes, whereafter the cells were washed in FACS buffer (PBS containing 0.1% sodium azide and 1% FCS) before staining with a combination of the following rat anti-mouse antibodies PE-Cy7-, PerCP-Cy5.5-anti-CD8α (53-6.7, RM4-5), APC-Cy7-anti-CD4 (GKI.5), FITC-, PE-Cy5.5-anti-CD44 (IM7), FITC-anti-CD45RB (16A), PE-Cy5-anti-CD11a (2D7), FITC-anti-CD107b (ABL-93), FITC-anti-CD107a (ID4B), PE-, APC-anti-CD62L (MEL-14) all purchased from BD Pharmingen (San Diego, USA), R&D systems (Minneapolis, USA) or eBiosciences (San Diego, USA). Cells were washed with FACS buffer before fixation and permeabilization using the BD Cytofix/Cytoperm™ (BD, San Diego, CA, USA) according to the manufacturer's protocol before staining intracellularly with PE-, PE-Cy7, APC-Anti-IFN-γ (XMG1.2), PE-anti-TNFα, and/or PE-, APC-anti-IL-2 (JES6-5H4). When FITC-anti-CD107b (ABL-93) and FITC-anti-CD107a (ID4B) were used these were added to the wells along with the antigens at the beginning of the incubation period. Furthermore, a PE-conjugated Pro5® MHC-I (*H-2K^b^*) pentamer (Proimmune, England) loaded with the minimal CD8 epitope of TB10.4 was used. Due to technical issues, the MHC-I molecules of the pentamer was loaded with TB10.4 _4–11_ instead of TB10.4 _3–11_. After washing, cells were resuspended in formaldehyde solution 4% (w/v) pH 7.0 (Bie & Berntsen, Denmark) and samples were analysed on a six-colour BD FACSCanto flow cytometer (BD Biosciences, USA). Data analysis was done with FACSDiva Software (Becton-Dickinson, San Diego, CA, USA) and Flowjo Software (© Tree Star, Asland, OR, USA). To calculate the %CD4/10.4 or CD8/10.4 T cells out of all lymphocytes the following equation was used (shown here for the CD4/10.4 T cells): %IFN-γ^+^ CD4 out of CD4 T cells×((%CD4 T cells*/(%CD4 T cells*+%CD8 T cells*), *out of all counted lymphocytes))×100%).

### In vivo cytotoxicity assessed by adoptive transfer of CFSE-labeled target cells

Single cell suspensions of CB6F1 spleens were obtained by passage through a fine metal mesh filter. Erythrocytes were depleted by lysis in ammonium chloride solution, washed in PBS before resuspension in incomplete RPMI and stained with 5(6)-Carboxyfluorescein diacetate *N*-succinimidyl ester (CFSE)(Sigma-Aldrich, San Louis, USA) at CFSE^High^ (20 µM) or CFSE^Low^ (2 µM) concentration for 10 min at 37°. Excess CFSE was quenched with RPMI containing 10% FCS and subsequently washed in medium without FCS. Next, CFSE^High^ labeled cells were pulsed with TB10.4 _3–11_ or TB10.4 _74–88_ at a concentration of 10 µg/ml of peptide for 1.5 hr at 37°. After being washed and resuspended in PBS the CFSE^High^ and CFSE^Low^ suspensions for each peptide was mixed at equal volumes. Final solutions of 20×10^6^ cells in a volume of 200 µl were given intravenously into naïve and infected mice. 18 hrs later adoptively transferred mice were sacrificed. Lungs were removed, homogenized and resuspended in formaldehyde before acquisition on a BD FACSCanto flowcytometer (BD Biosciences, USA). To evaluate the frequency of specific lysis, the ratio of CFSE^High^ and CFSE^Low^ of infected mice were compared to naïve control mice and was calculated using the formula (1−(%CFSEhigh cells/%CFSElow cells) ×100%). For the infected and naïve groups 3 and 2 mice were used respectively.
